# Harm reduction services for British Columbia's First Nation population: a qualitative inquiry into opportunities and barriers for injection drug users

**DOI:** 10.1186/1477-7517-3-30

**Published:** 2006-10-11

**Authors:** Dennis Wardman, Darryl Quantz

**Affiliations:** 1 Department of Health Care and Epidemiology, University of British Columbia, Vancouver, British Columbia, Canada; 2 Vancouver Coastal Health Authority, Vancouver, British Columbia, Canada

## Abstract

**Background:**

Aboriginal injection drug users are the fastest growing group of new Human Immunodeficiency Virus cases in Canada. However, there remains a lack of comprehensive harm reduction services available to First Nation persons, particularly for First Nation people dwelling in rural and reserve communities. This paper reports findings from an exploratory study of current harm reduction practices in First Nation communities. The purpose of this study was to provide an overview of the availability and content of current harm reduction practices, as well as to identify barriers and opportunities for implementing these services in First Nation communities.

**Methods:**

Key informant interviews were conducted with 13 addictions service providers from the province of British Columbia, Canada.

**Results:**

Participants identified barriers to these services such as community size and limited service infrastructure, lack of financial resources, attitudes towards harm reduction services and cultural differences.

**Conclusion:**

It was recommended that community education efforts be directed broadly within the community before establishing harm reduction services and that the readiness of communities be assessed.

## Background

Over the last decade there has been growing concern in the public health sector over the spread of Human Immunodeficiency Virus (HIV) in Canada's First Nation communities. Much of this concern has been directed towards Aboriginal injection drug users (IDUers), which is the fastest growing group of new HIV cases in Canada [1]. Unfortunately, there continues to be a lack of recognition and awareness of HIV and its risk factors in Aboriginal communities [1,2]. Concurrently, there remains a lack of comprehensive harm reduction services available to First Nation persons, particularly in rural and reserve communities [2-4]. There is significant diversity in British Columbia's First Nation communities with over 198 separate bands in all geographic locations of the province. Many of these communities are small and isolated and have limited access to many health services. British Columbia's First Nation population currently suffers from higher mortality rates from illicit drug use and related health conditions [3] and the frequent movement between reserve (rural) and urban areas poses a further threat of HIV infection to non-IDU users [5,6]. An effective response to these conditions is needed urgently for Aboriginal communities.

The creation of culturally appropriate and effective harm reduction (HR) services and policies has been advocated as a necessary response to combat the increasing rates of HIV among First Nation persons. A requisite to this process is a clear understanding of the perceptions and barriers around HR services in First Nation culture and communities. Community responses to addictions are often directed at more prevalent issues such as alcoholism and tobacco. Further, most HR services are located in larger urban areas, and are more likely to be targeted at the general population. Many First Nation persons are not comfortable within the health care system and may not access non-Aboriginal services. This study was an opportunity to explore HR services for injection drug users located within British Columbia's First Nation communities. The objectives of this exploratory study were to: 1) provide an overview of the availability and content of current HR practices; 2) identify barriers for HR services, and; 3) outline potential opportunities and recommendations for creating and facilitating HR practices in First Nation communities.

## Methods

### Participant recruitment

A purposive sampling strategy was utilized to recruit participants for this study. In June 2003, a letter from the first author was sent out to major service organizations and agencies that included within their mandate HR services for First Nation IDUers. These agencies were asked to participate in the study through the nomination of interview candidates who had experience in the issues surrounding IDU-related HR services in First Nation populations, either through program planning or service delivery. From this strategy, 13 individuals were recruited to participate in an interview to discuss their experiences and perceptions around HR. All of the agencies invited to participate were located within the province of British Columbia. Ethical approval for the study was obtained from the Office of Research Services at the University of British Columbia.

### Data collection and analysis

The study adopted a qualitative approach and consisted of a series of in-depth interviews. To facilitate this process, an interview guide was developed and included questions regarding the availability of HR services for First Nation persons; content of current HR programs; barriers to these services; and perspectives on opportunities for creating and establishing these services. Interviews were conducted either face-to-face or by telephone over a two-month period and lasted between 30 and 60 minutes each. The first author is an Aboriginal person and conducted all of the interviews. Written consent was obtained from all participants and interviews were audio taped and transcribed into a word processor.

Initial analysis of the data was undertaken simultaneously with data collection. Through this review, emerging themes were sought and additional prompts and questions were identified for future interviews. The formal analysis process began with an independent review by both authors of all transcripts, during which units of data were assigned codes based on themes or issues. Codes are "tags or labels for assigning units of meaning to the descriptive or inferential information compiled during a study and are used to retrieve and organize data" [7]. The next stage of data analysis involved the task of categorical aggregation [8]. In this process, the coded data are reviewed to collect similar instances in an effort to elicit common themes and create a framework to answer the research questions. This process was facilitated through the use of various tools such as charts, matrices and memos [7]. Emerging themes were explored between the interviews in an effort to search for relationships, consistencies and/or inconsistencies. Both authors met regularly to compare and contrast analysis results in order to ensure that all themes were captured. Credibility was assessed through member checks, a technique in which the findings and interpretations of the researcher are taken to informants for verification [8]. In this case, a transcript of the interview and a summary of the final themes were taken back to participants for feedback and verification.

## Results

This study yielded a number of perspectives and recommendations around the availability and provision of HR services in Aboriginal communities. Service providers valued the opportunity to share their experiences and opinions on how these services can be more effective, either independently or in conjunction with other services in the community. Participants also described what they felt have been barriers to the development of these services, as well as issues around clients access to HR. The results are described below.

### Service content and delivery

It was clear from the interviews that both the message and content of any HR services must be appropriate for the client. The incorporation of traditional Aboriginal practices was seen as the most important element of developing and providing HR services. Aboriginal cultural practices were viewed as having several strengths that were beneficial to the clients.

"Culture is a strength in that it provides a different way of doing things in life, like say a powwow, you have drums, you have dance, you have physical exercise, you have a sense of belonging...these are the times that bring people together and in these places drugs and alcohol are not tolerated."

Part of these traditional practices involves the use of oral tradition, and this was reflected in participants' positive comments about allowing clients to share stories of their experiences. This sharing was viewed as an opportunity for clients to learn and think about how can they apply to their own lives what they have learned through the services.

"Hearing people's personal stories, who have been addicted, or living with different illnesses...they have more compassion and more willing to listen and understand other ways of doing things."

In addition to incorporating and respecting cultural practices, programs must also respect both age and literacy levels of clients. For example, younger clients may prefer a more direct style of communication and/or the older generation may find it inappropriate to discuss these issues more directly.

"The youth prefer that you be blunt with them, and take it to their level of language. The youth have a certain way of communicating with each other...tell them the realities of their action."

Providing additional services in conjunction with HR programs was also viewed as an opportunity for more successful program development and delivery. For example, providing only a needle exchange program without additional education, counseling and other health services, was viewed as an ineffective practice.

"Harm reduction services have to go along with education and awareness interventions for the client...they need support, not just needles."

Harm reduction services must attempt to integrate into new and/or existing reserve programs and could be enhanced by providing at least some training to all service staff, even if they are not directly working in HR services.

Quote A:" It seems many front-line workers have a lack of understanding and receive no training around HR."

Quote B:" Agencies that service the front-line workers, they've been really low key around HR, they can really influence front-line workers."

Finally, comments were made regarding the use of Aboriginal staff. Although Aboriginal staff were viewed as being beneficial for building trust with some clients, others thought that working with non-Aboriginal staff would not be a major concern for many clients, especially if service providers are both open minded and non-judgmental.

### Availability

Participants described a number of issues around IDUer access to HR services. Perhaps, most challenging was the lack of availability of comprehensive HR programs, both in and around First Nation communities. In the province of British Columbia there are nearly 200 First Nation communities, many of which have small populations. Hence, the provision of any service, especially for the smaller communities, can become difficult. For example, several participants noted that there are only a few informal needle-exchange programs that actually exist within First Nation communities. Participants noted that it is easier for larger communities to find ways to provide these services.

"Small communities with only 1 or 2 workers have more difficultly to do deliver HR services...Larger communities can piggy-back HR services into existing services."

Aboriginal communities with small populations often utilize the services of neighbouring towns and cities. Unfortunately, these services may not even be available in these centers. Many communities do not want these services for fear of attracting IDUers. Those seeking services may be forced to travel long distances to larger urban centers in order to access HR services:

"A client had to drive 6 hours to a pharmacy to pick up Methadone, so what chance of success is there?"

### Barriers

Participants discussed and identified two major issues that they felt were barriers for clients in using HR services, particularly in First Nation communities. The first of these issues revolved around the stigma attached to both addictions and, concurrently, the use of HR services by those with addictions. Clients may be shunned because the attitude in many First Nation communities is that of an abstinence model for the treatment of addictive disorders. Participants noted that many addictions counselors working in First Nation communities are dealing with past addictions issues and often advocate for abstinence; therefore they condone continued use of substances even if use leads to less harm reduction for the client. Furthermore, First Nation cultural beliefs may also be at odds with the HR model that 'allows' individuals with addictions to continue abusing drugs.

"The traditional cultural approach is that First Nation substance abuse is not the norm and it shouldn't be. So it's approached from the abstinence point of view."

Unfortunately, another concern that was voiced was a feeling of helplessness and frustration in serving certain clients. Accessing HR services has a stigma of failure attached to it and is viewed by some as the last option after other treatment modalities have failed. For many service providers and community members, there is a sense of giving up when addicts continue to abuse substances.

"Basically clients accessing HR services are seen as at the end of services. They've tried treatment, it didn't work, and they tried counseling it didn't work. So just give them the Methadone and keep them safe, just give them needles. It's almost like they are written off."

Another barrier for clients identified by participants was the fear of a lack of confidentiality when they access these services. Whether this fear is real or not is unclear but some clients are reluctant to access services within their community for fear of being identified.

"The Aboriginal community is small so they don't want aboriginal staff who they'll likely know, and then they'll know that they are an IDUer."

Finally, another barrier may also be a lack of support for HR services in terms of policies or management. A reluctance to support HR services from boards or management due to political or philosophical reasons were noted as an issue for many communities. Again, a stigma attached to providing services to those who continue their addiction may contribute to this.

Quote A: "Boards for NNADAP Centres are reluctant to develop policies to accept HR clients. They follow the abstinence approach."

Quote B:" I have spoken with nurses who are pushing for a needle-exchange programs because they know there are a high number of IDUers in their community but leadership doesn't want outsiders to think the whole reserve is crumbling, have a heavy drug use problem. So to get around this, nurses have offered needle-exchange programs to everyone, such as Diabetics. This way, no one gets singled out."

### Education

Participants had several recommendations to enhance the profile and availability of HR services. The key component of these recommendations was focused around education to make all sectors of the community more aware of the need for these services. It was felt that there is a general lack of awareness around how HR services can fit with existing philosophies and treatments of addictions.

Quote A:" The need for community HR education, including what HR is, why the need for it, its potential impact, and how fits in with abstinence model of treatment through the use of personal stories and presentation in a fashion that a community is familiar with."

Quote B: "The whole HR strategy needs to be normalized, its seen as in opposition to abstinence, got to be better informed around how this is part of the continuum of services for addicts...People need a visual representation of HR to see how it fits in with treatment."

Participants suggested that educational efforts should be done in a participatory fashion in order to capture the experiences of those who would be affected by the integration of HR services. Another suggestion was to combine HR education with other health promotion initiatives that might provide an easier opening.

Quote A:" ...In our workshops, we brought in street nurses so people could share exactly how it's done. Talking about their experiences normalizes HR."

Quote B: "Start off with something familiar like Diabetes and discuss what HR services are in context of Diabetes.... or start with tobacco and then into IDU. Something that people won't be scared of right away."

Participants also strongly emphasized the diversity of groups that must be targeted in education campaigns. The value of community leaders must not be under estimated, as their support is crucial for delivering HR services. In addition to elected leadership, this group also includes gaining the support and trust of community Elders who can also play a key role in advocating for these services.

"I think when you talk with an elder, they're hesitant at first but once they learn the issues around the diseases, then they're willing to help because they know their responsibility as an elder, to teach others and to be understanding."

Although the impact of education on key community leaders was emphasized, participants also noted that efforts must also be directed towards the community at large. Changing community members' attitudes and beliefs around HR was seen as vital in gaining widespread acceptance of HR services.

"The more people educated as to what HR is, the greater the impact it will have down the road."

Community education efforts could be facilitated by existing media, which are often used for communication in First Nation communities. These include mass media, printed media and community newsletters. Communications must be well planned and appropriate for the audience in order to create a discourse and lasting change around this issue.

"Most communities have a newsletter, which would be great to get the message out. Mass media would be helpful. We need a marketing strategy to get the message out because it's becoming more pervasive, more prevalent, and will have more consequences once it starts getting talked about."

Finally, participants noted the lack of research information around the needs and access to HR services in First Nation communities, particularly for rural areas. It was suggested that IDU is often not recognized as a problem in communities. Many participants felt that further research knowledge and dissemination is a key asset in these education efforts. In addition, this knowledge would provide learning tools and guidance in the development and administration of First Nation HR programs. Some participants noted the frustration of many frontline workers in trying to change attitudes towards HR within their communities. They suggested that information coming from trusted credible professionals might be more successful in gaining acceptance.

"The message has to come from medical experts, mental health experts, to the grassroots down."

## Discussion

The establishment of HR services in Aboriginal communities has proven to be a challenge on a number of fronts. Issues such as community size and limited service infrastructure, lack of financial resources and cultural differences are some of the potential barriers to initiating these services. This study makes a unique contribution to the literature by exploring the issue of HR from the perspective of care providers in Aboriginal communities who are directly and/or indirectly involved in HR services. As such, this information will be of value to policy makers, Aboriginal leaders and program planners with an interest in establishing these services.

The results of this study overwhelmingly highlight the need for both enhanced and culturally appropriate HR services. This is particularly vital, given that the provision of culturally appropriate education sessions can enhance participant satisfaction and retention [9]. The availability of these services was raised as a particular concern, considering the small size of many Aboriginal communities. A potential strategy for this problem includes partnering with existing services. A multi-faceted educational approach was seen as a requisite for facilitating the establishment and provision of HR services. Participants outlined the need for educating the general community, bandleaders, other health care providers and the involvement of Elders in this process.

Participants emphasized the need for community buy-in for all aspects of HR. This was noted as a particular concern, considering that the idea of HR did not exist in traditional Aboriginal culture, nor was there a concept of addiction since mind-altering substances were used in a controlled fashion for ritualistic purposes [10]. There thus emerges the ethical issue of imposing the practices and inherent values of one culture, in case through a HR philosophy, onto another. First Nation peoples have their own traditional beliefs around the prevention and treatment of illness. For example, in a study among Aboriginal participants attending a Diabetes education program, most participants believed that Aboriginal people had their own way of treating Diabetes and one-third believed traditional medicine could cure the disease [11]. This suggests the need for sensitivity in when exploring the establishment of HR services.

One potential approach for First Nation communities considering HR services may be to present educational information with the acknowledgement that cultural differences exist. Communities will then be in a more informed position to decide whether HR services should be established. If a community is reluctant to consider HR services, it may be unwise to proceed. A disregard for one's personal and healing beliefs could impact that person's attitude towards HR (and other services), as well as his or her understanding of and compliance with therapeutic regiments [12]. Considering the diversity among British Columbia's first peoples, further research into this area is needed to determine best practices for incorporating traditional beliefs into HR services.

## Competing interests

I (Dennis Wardman) declare that I have no competing interests.

I (Darryl Quantz) declare that I have no competing interests.

## Authors' contributions

DW conducted all of the interviews. DQ and DW participated equally in all elements of the data analysis process and the writing and approval of the final manuscript.
